# A GC-MS-Based Metabolomics Investigation of the Protective Effect of Liu-Wei-Di-Huang-Wan in Type 2 Diabetes Mellitus Mice

**DOI:** 10.1155/2020/1306439

**Published:** 2020-08-13

**Authors:** Jian-hua Huang, Dan He, Lin Chen, Qing Du, Rong Yu, Ping Cai, Shui-han Zhang

**Affiliations:** ^1^Hunan Academy of Chinese Medicine, Hunan University of Chinese Medicine, Changsha, Hunan 410013, China; ^2^Hunan Key Laboratory of TCM Prescription and Syndromes Translational Medicine, Hunan University of Chinese Medicine, Changsha, Hunan 410208, China

## Abstract

**Materials and Methods:**

MKR mice were used for the development of diabetes with high-fat diet feeding. These mice were further injected with streptozocin (STZ) to aggravate kidney failure. Fasting blood glucose (FBG) and urinary albumin-to-creatinine ratio (ACR values) were determined to validate the successful establishment of diabetic models with desired kidney dysfunction. Metabolomics approach coupled with gas chromatography-mass spectrometry (GC-MS) and random forest (RF) algorithm was proposed to discover the metabolic differences among model group and control group as well as to examine the therapeutic efficacy of traditional Chinese medicine, Liu-Wei-Di-Huang-Wan (LWDHW), in diabetes and associated kidney failure.

**Results:**

Some metabolites such as 3-hydroxybutyric acid, citric acid, hexadecanoic acid, and octadecanoic acid showed significant differences between the control group and model group. Treatment with LWDHW resulted in a significant decrease in FBG and ACR values. These results suggested that LWDHW could have beneficial effects in diabetes-associated renal failure.

## 1. Introduction

Type 2 diabetes mellitus (T2DM) is known as a chronic multifactorial disease, characterized by metabolic, hormonal, epigenetic, and oxidative imbalance [1, 2]. The main pathological features of T2DM include chronic hyperglycaemia and dyslipidaemia. However, the risk of T2DM is associated with various complications, such as retinopathy, nephropathy, neuropathy, ischemic heart disease, and peripheral vasculopathy [1, 2]. Epidemiological evidence suggested that diabetic kidney disease (DKD) is one of the most severe diabetic microvascular diseases and a leading cause of end-stage renal disease [3, 4]. Approximately 20–40% of diabetic patients will ultimately develop DKD, which is also associated with high risks of cardiovascular morbidity and mortality [5, 6].

Liu-Wei-Di-Huang-Wan (LWDHW) is a classical traditional Chinese formulation, having clinical efficacy for “nourishing kidney-yin”. It was first mentioned during the Song Dynasty (AD 1119) by Qian Yi in his book “*Pediatric Medicinals and Patterns*” [7, 8], since then it has been used for the treatment of T2DM [9] for thousand years in China. It is known to effectively decrease rat FBG values [10] and can also attenuate deterioration of albuminuria in type 2 diabetes patients, when coadministered with Ginkgo Biloba tablets [11]. This formulation consists of six commonly used Chinese herbs: (1) *Shu Di Huang* (*Rehmannia glutinosa* Libosch), (2) *Shan Yao* (*Cornus officinalis* Sieb. et Zucc), (3) *Shan Zhu Yu* (*Cornus officinalis* Sieb. et *Zucc*), (4) *Mu Dan Pi* (*Paeonia suffruticosa* Andr.), (5) *Ze Xie* (*Alisma orientalis* (Sam.) Juzep.), and (6) *Fu Ling* (*Poria cocos* (Schw.) Wolf), in the ratio of 8 : 4 : 4 : 3 : 3 : 3, respectively.

Mounting evidences have demonstrated that this formulation is also effective in maintaining neuroendocrine immunomodulation balance, improves cognitive function [12, 13], and delays the progression of renal failure [14]. Chen et al. have reported that LWDHW can induce CYP1A2, suppress CYP2A6 and NAT2 activities, and affect caffeine metabolism in *in vivo* model [15]. Cheng et al. have recently found that LWDHW can also help in controlling the plasma glucose levels [16]. Hsu and coworkers reported the beneficial effects of LWDHW on kidney patients. It is also known that integrating TCM healthcare into diabetes care can be associated with decreased risk of developing kidney failure [17].

Metabolomics is a systematic study of metabolic changes of small molecules in response to the changes of both endogenous and exogenous factors and has many potential applications and advantages for the research of complex systems, such as studies of metabolic diseases and therapeutic effects of medicines [18–23]. Diabetes and DKD are typical heterogeneous metabolic disorders, characterized by abnormal metabolism of carbohydrates, lipids, and proteins; thus, metabolomics seems to be a powerful tool for evaluating disease process and for identifying the mechanism of action of currently used therapeutic agents. Therefore, in this study, we exploit a metabolomics approach to investigate the effects of TCM formulation, Liu-Wei-Di-Huang-Wan, in controlling of FBG and prevention of renal failure.

## 2. Materials and Methods

### 2.1. Chemicals

Streptozocin (STZ), bis-(trimethylsilyl)-trifluoroacetamide (BASFA) with 1% trimethylchlorosilane (TMCS), pyridine, and methoxyamine hydrochloride, 2-isopropylmalic acid, and heptadecanoic acid (internal standard) were purchased from Sigma-Aldrich (St. Louis, MO, USA). *Liu-Wei-Di-Huang-Wan* (Batch no. 20180402, 360 pills/bottle, 0.14 g/8 pill) was purchased from Hunan JIU-ZHI-TANG Co., LTD (Changsha, Hunan, China). Gliquidone (Batch no. 114023, 30 mg × 24 pills) was purchased from Beijing Wanhui Double Crane Pharmaceutical Co., Ltd.

### 2.2. Sample Preparation

The drug production standard was that *Mu Dan Pi (Paeonia suffruticosa* Andr.) was primarily extracted by using a water-vapor volatile oil extraction unit. The volatile oil was collected and the residue was mixed with other five kinds of herbs, immersed in distilled water (1 : 8, w/v) for 0.5 h, and extracted twice by refluxing with boiling water for 2 h. All of the filtrates were mixed together and vacuum concentrated and then mixed with volatile oil to form pill. 1 g of sample was weighted and dissolved in 25 mL methanol (50%), and then the mixture was filtered with 0.45 *µ*m for HPLC analysis.

### 2.3. HPLC Analysis

An Agilent-1260 HPLC system (Agilent Technologies, MA, USA) equipped with a quaternary pump and UV detection system along with Agilent Poroshell 120 SB-C18 (250 mm *∗* 4.6 mm, 5 *µ*m) column was used to analyze samples. The detection wavelength was set at 235 nm, with a flow rate of 0.2 ml/min, and the column temperature was 25°C. The mobile phase was composed of acetonitrile (solvent A) and 0.1% formic acid (solvent B). The gradient procedure was set as 2% A at 0–2 min, 2–22% A at 2–25 min, 22–35% A at 25–32 min, and 35% A at 32–40 min. The injection volume was 0.2 *µ*L.

### 2.4. Animal Models

Mice overexpressing a dominant-negative IGF-1R specifically in skeletal muscle (MKR mice) were first established by Fernandez and coworkers [24]. This model showed insulin resistance and can rapidly develop into overt diabetes. Then, this model was successfully used in subsequent studies [25, 26]. In the current study, forty MKR mice (12 weeks old) were randomly divided into four groups: MKR mice group, model control group, LWDHW group, and western medicine group (gliquidone), 10 mice for each group. However, ten C57BL/6 mice were selected as controls. MKR mice group, LWDHW group, and western medicine group were injected with 1% streptozocin (STZ) dissolved in citric acid buffer (pH = 4.5) at a dose of 50 mg/kg/day, for 5 days, and the model control group and the control group were injected with the same dose of citric acid buffer. However, the control and the model control group were fed with a routine diet, and a high-fat diet was given to other groups for 4 weeks. Fasting blood glucose (FBG) values and urinary albumin-to-creatinine ratio (ACR) were tested to determine the development of diabetes in desired groups.

LWDHW and western medicine groups were treated with Liu-Wei-Di-Huang-Wan (1.08 g/kg/d) and gliquidone (7.80 mg/kg/d), respectively (once a day for 28 days). On 29^th^ day, mice were sacrificed, and blood samples were collected by cardiac puncture. FBG values and urinary ACR were determined. The protocols in this experiment were approved by the Animal Ethical Committee of the Hunan University of Chinese Medicine. All experimental procedures were conducted in accordance with the Guideline for the Care and Use of Laboratory Animals.

### 2.5. Collection of Serum Samples and Pretreatment

Venous blood was collected from fasting mice in a blank tube without anticoagulant or preservative. The fresh blood was stored at 4°C for 1 h, and serum was collected by centrifugation at 3000 rpm and stored at −80°C until further use. Before analysis, the serum was thawed at 4°C for 30 minutes.

Firstly, 10 *µ*L blood was collected from each sample to conduct quality control (QC) sample and vortex-mixed for 60 s, and each 100 *µ*L blood was extracted to form different QC samples for daily analysis. In this way, QC samples can be used to validate the stability of equipment and the performance of the proposed method.

Secondly, 100 *µ*L blood sample was extracted from each sample including QC samples and vortex-mixed with 300 *µ*L methanol (including 1 mg/mL of heptadecanoic acid/methanol as internal standard) for 15 s, and this mixture was further centrifuged for 15 min (14,800 rpm, 4°C) to remove proteins. The supernatant (370 *µ*L) was freeze-dried and mixed with methoxamine/pyridine (20 mg/mL) for 15 s and incubated for 1 h at 70°C, followed by the addition of 100 *µ*L of BSTFA, and incubated again for 1 h (70°C). The supernatant was used for GC-MS analysis. Finally, after being preprocessed, all the samples were analyzed by using GC-MS at random order, and after the 5 samples were analyzed, QC sample was injected once to validate the stability of the equipment. QC samples analyzed on different days can be used to validate the reproducibility of sample preparation.

### 2.6. Gas Chromatography-Mass Spectrometry Conditions

Metabolite analysis was performed on a DB-5MS capillary column (30 m × 0.25 mm × 0.25 *µ*m) in Shimadzu GC-MS system. The detailed parameters and conditions of GC-MS analysis were similar as reported previously by our group with slight modifications [27]. The temperature was maintained at 70°C for 4 min and then programmed to rise to 300°C at a rate of 8°C/min and held for 3 min. The temperatures of the front injection port, ion source, and interface were set at 260°C, 280°C, and 230°C, respectively. The flow rate of helium gas was 1.0 mL/min. One microliter of the sample was injected at a ratio of 10 : 1 split mode. The mass spectrometer was operated under electron impact (EI) mode at ionization energy of 70 eV and 0.90 kV detector voltage in 0.2 s/scan for full scan. The mass spectrometer was selected with *m*/*z* ranging from 55 to 600.

### 2.7. Data Processing and Analysis

Metabolites in these GC-MS profiles were identified by using their chromatographic and mass spectral characteristics. The identification of metabolites and internal standard was based on the search results in the National Institute of Standards and Technology (NIST 07) mass chromatography library in the GC-MS Postrun Analysis software (Shimadzu). Metabolites were also validated by comparing with references' results with retention time and mass spectra. The internal standard was used for normalization in relative quantitative analysis of these metabolites. The raw data acquired by the analytical instruments were firstly processed with peak detection and alignment. Subsequently, the processed data were imported into Matlab software 8.0.1 (Mathworks, USA), and random forest method was used to discover the metabolite differences among different groups and further to identify the most valuable metabolite for distinguishing different groups. MetaboAnalyst 3.0 was used for biological pathway analysis based on these metabolites' information (http://www.metaboanalyst.ca/) [28].

## 3. Results

### 3.1. Qualitative Analysis of Bioactive Compounds in Liu-Wei-Di-Huang-Wan

The high-performance liquid chromatography-ultraviolet (HPLC-UV) method was used for the quality control of LWDHW. Five major constituents, including loganic acid, loganin, 6′-O-galloylpaeoniflorin, benzoylpaeoniflorin, and paeonol, were identified, as depicted in Figure S1. The contents of these five compounds were 0.18 mg/g, 2.53 mg/g, 0.22 mg/g, 0.65 mg/g, and 1.62 mg/g, respectively, and more detailed information can be found in Tables S1 and S2.

### 3.2. Assessment of MKR Model and Therapeutic Effects of LWDHW

Three animal groups were established, i.e., normal control group (healthy mice), model control group (diabetic mice), and MKR group (diabetic mice with kidney injury). As MKR mice can develop into overt diabetes by feeding with a relatively high-fat diet, MKR group was further injected with STZ to induce kidney injury. FBG values and urinary albumin-to-creatinine ratio (ACR) values of each group were determined to estimate the animal model, as listed in Table 1. As can be seen from Table 2, the FBG and ACR values for the model control group (diabetic mice) are higher than those of the normal control group (healthy mice), while the FBG and ACR values of the MKR group (diabetic mice with kidney injury) are higher than those of the model control group (diabetic mice). This means the FBG and ACR values can exhibit the states of disease.

After treatment, the FBG value for the LWDHW group was decreased from 14.12 to 9.509; similar results were observed in the western medicine group, which showed a decrease in FBG value from 14.12 to 8.718. The FBG values for the model group (MKR group) did not show a significant decrease. These results indicated that both western medicine and traditional Chinese medicine could reverse the symptoms of diabetes in mice as well as reduce the impact of a high-fat diet. The urinary albumin-to-creatinine ratio (ACR) was also calculated for each group. ACR values for the LWDHW group were decreased from 150.8 to 115.8, while a decrease in ACR values from 151.2 to 112.4 was also observed in the western medicine group. These results were consistent with the FBG values, and these results showed the drugs had prevention effects to the kidney.

### 3.3. GC-MS-Based Metabolomics Method

#### 3.3.1. Reliability of GC-MS-Based Metabolomics Method

Internal standards and QC samples were used to assess the stability and repeatability of the current method. In the daily analysis process, three QC samples were initially injected for calibrating GC-MS equipment followed by injection of two internal standard samples. Response from both internal standard and QC samples was used to validate the stability of equipment at different analysis times. QC samples were also injected at different intervals during the experiment (after five samples). The results of these QC samples were used to monitor the stability of the whole analytical system as well as the reproducibility of sample preparation.

The variations of internal standards and QC samples (except the first three QC) were calculated. RSD values for metabolites ranged from 1.32% to 12.30%. Moreover, the RSD values of two internal standards, 2-isopropylmalic acid and heptadecanoic acid, were 2.3% and 2.9%, respectively. These results showed that the current method has good stability and reproducibility. The detailed results for QC samples are listed in Table S3.

#### 3.3.2. Endogenous Metabolites Identified by GC-MS

A total of 30 endogenous metabolites were identified, and the representative total ion chromatogram of each group is presented in Figure S2. Detailed information about the identified metabolites, including compound name, retention time, and quantitative ions, is listed in Table 2.

### 3.4. Metabolites' Analysis between MKR Group and Model Control Group

In this section, we aimed to find the differences among the MKR group, model control group, and normal control group from their metabolites' aspects and further identify some potential biomarkers for distinguishing different groups that can help in the diagnosis of DKD disease.

All normalized metabolites' profiles of the MKR group, model control group, and normal control group were analyzed by random forest method. Random forest method had been used previously in our research group and has showed its advantages in complex metabolic data analysis [29, 30]. It could effectively extract characteristic features from these metabolites' data and visually reveal the relationships among different groups.

**Figure 1:**
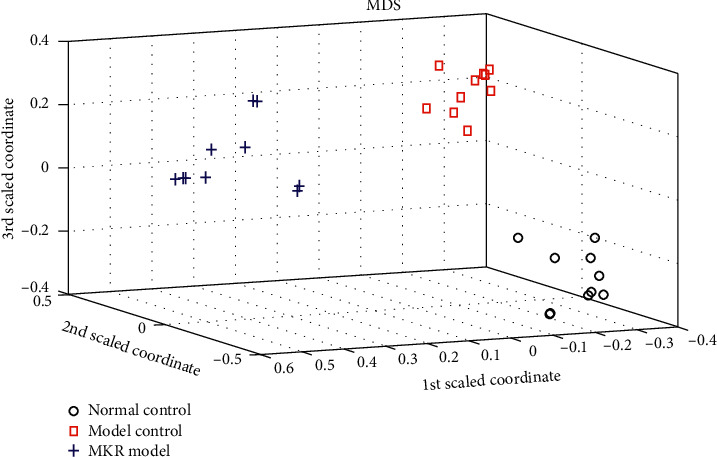
Classification plots of three groups by random forest algorithm.

The classification plot of three groups is shown in Figure 1. Three groups were well separated, and the differences among these groups were obviously observed. These results showed that metabolites' profiles can reveal the metabolic disturbances in DKD progress, which can also be used for the diagnosis of DKD. Therefore, RF algorithm was employed to classify samples from the MKR group and normal control group. The prediction accuracy, sensitivity, and specificity for the current method were 94.33%, 88.96%, and 91.57%, respectively.

**Figure 2:**
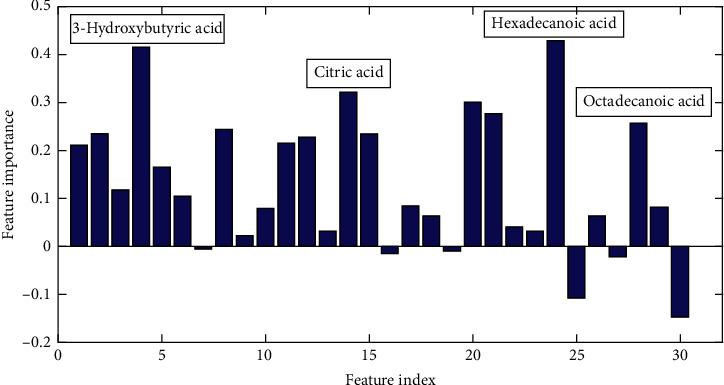
Variable importance of all the metabolites.

Furthermore, in the classification model establishing process, the contribution of each metabolite for distinguishing was calculated. As can be seen from Figure 2, some metabolites such as 3-hydroxybutyric acid, citric acid, hexadecanoic acid, and octadecanoic acid have higher contributions in distinguishing the MKR group from the normal control group. These metabolites with higher contributions were considered as potential biomarkers for the diagnosis of DKD.

**Figure 3:**
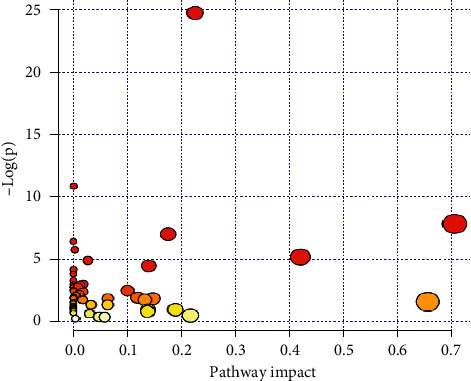
Biological pathways analysis based on all the metabolites.

Metabolic pathways of alanine, aspartate, and glutamate metabolism and linoleic acid metabolism play important roles in the progress of DKD (Figure 3). MetaboAnalyst 3.0 software was used to further evaluate the metabolic pathways associated with the identified metabolites. MetaboAnalyst 3.0 is one of the most popular software used in metabolomics data analysis, which used the high-quality KEGG pathway database as backend knowledgebase. Their impact indexes are 0.7056 and 0.6562, respectively. The detailed metabolic pathways informatics is listed in supporting information Table S4 and Figures S3 and S4, respectively.

### 3.5. Comparative Metabolites' Analysis between MKR Group and Treated Group

**Figure 4:**
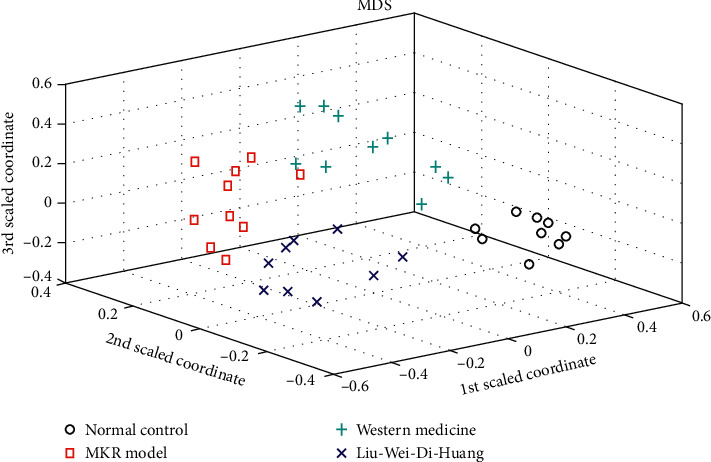
Classification plots of four groups by random forest algorithm.

Changes in metabolic, physiological, and pathological conditions induced after administration of Liu-Wei-Di-Huang-Wan and gliquidone were further analyzed. The cluster analysis results for the model group, LWDHW group, western medicine group, and normal control group are shown in Figure 4.

As shown in Figure 4, the normal control group is located in the right of the cluster analysis plot, while the MKR group is located in the left part of cluster plot. These results indicated an obvious difference in the metabolic patterns of DKD mice and normal group. Treatment with Liu-Wei-Di-Huang-Wan and gliquidone for 28 days improved the metabolic pattern of mice, as depicted in the cluster plot. Both the LWDHW group and western medicine group were closer to the normal control, which indicated a comparative therapeutic efficacy of LWDHW with gliquidone. Besides, the authors also found that the intensities of some metabolites were different among LWDHW and western medicine groups. LWDHW had better regulation effects on metabolites, such as oxalic acid, butyric acid, serine, D-galactose, D-glucose, D-arabinose, and linoleic acid, while gliquidone had better regulation effects on L-proline, glycine, L-threonine, erythronic acid, N-acetyl-D-glucosamine, D-mannose, L-lysine, and D-turanose. These results might be caused by the different therapeutic pathways of two medicines. More comprehensive and deep study would be implemented based on these metabolites' information. Thus, the advantage of metabolomics was that it can provide more information for the mechanism research.

## 4. Discussion

It is generally considered that TCMs exert a synergistic action to achieve the therapeutic benefit; the synergies may contain a series of complex interactions due to the coexistence of multiple components. It is difficult to explain all the effects caused by these components. Metabolomics approach provided a suitable perspective to monitor the comprehensive physiological and pathological changes caused by disease and treatment. Metabolites are the final products of various actions from genomics to proteomes and so on. Thus, metabolomics is a research frontier of systems biology, which provides a new perspective to understand diseases and drug actions by identifying the signature metabolites that represent the global biochemical changes in living systems.

Previous studies have reported the presence of unusually high lipid deposition in the CKD and DKD patients, which suggest that excessive fatty acid (FA) accumulation and lipotoxicity can be a potential cause of kidney dysfunction. FAs are carboxylic acids with saturated or unsaturated aliphatic tails obtained from de novo synthesis or hydrolysis of triglycerides. They are primary substrates in energy metabolism, synthesis of membrane phospholipids, and bioactive compounds [31, 32]. An increased level of urinary FAs was found in DN patients, and these high levels were found to be in positive correlation with renal tubulointerstitial injury [33, 34].

3-Hydroxybutyric acid is synthesized in the liver from acetyl-CoA and can be used as an energy source by the brain when the blood glucose level is low. Blood levels of 3-hydroxybutyric acid may be monitored in diabetic patients as an indication of diabetic ketoacidosis. One of the functions of 3-hydroxybutyric acid is to provide acetoacetyl-CoA for the synthesis of cholesterol, fatty acids, and complex lipids [35]. In this study, we observed elevated levels of hexadecanoic acid and octadecanoic acid in the DKD progress. In the pathological status, the increased albumin-bound FAs are reabsorbed by proximal tubular epithelial cells (PTECs) and promote FA deposition in the kidney [36, 37].

## 5. Conclusion

In the present study, we employed metabolomics method to accurately differentiate the model group and control group and further represent the metabolic profile changes treated by TCM with high classification accuracy. These results demonstrated that metabolomics could be effectively used to mine the information hidden in the complex metabolomics data and hence can pave the way for the interpretation of these data. Therefore, metabolomics is an efficient and attractive alternative technique for diseases' diagnosis, pathogenesis, and pharmacodynamics research.

## Figures and Tables

**Table 1:** FBG and urinary ACR values of different groups before and after treatment.

Groups	FBG before treatment (mmol/L)	FBG after treatment (mmol/L)	Urinary ACR before treatment (mg/g)	Urinary ACR after treatment (mg/g)
MKR group	14.99 ± 2.022	13.95 ± 2.772	154.4 ± 6.976	185.2 ± 11.04
Model control group	7.855 ± 0.723	8.218 ± 0.9347	62.24 ± 6.201	83.04 ± 6.05
Liu-Wei-Di-Huang group	14.12 ± 1.656	9.509 ± 0.5752	150.8 ± 7.368	115.8 ± 6.968
Western medicine group	14.12 ± 1.224	8.718 ± 0.5307	151.2 ± 6.319	112.4 ± 7.232
Normal control group	5.145 ± 0.3417	5.209 ± 0.7355	5.382 ± 1.369	5.591 ± 1.436

**Table 2:** Metabolite information of each group after 28 days of treatment.

Id	TR (min)	Endogenous metabolites	Quantitatively results					HMDB
			MKR group	Model control group	Liu-Wei-Di-Huang group	Western medicine group	Normal control group	
1	6.563	Oxalic acid	1.0081 ± 0.0647	1.1050 ± 0.0638	1.2981 ± 0.198	0.996 ± 0.126	1.2671 ± 0.0878	HMDB 00190
2	7.329	L-Lactic acid	0.1151 ± 0.0176	0.0670 ± 0.0423	0.1443 ± 0.065	0.0831 ± 0.0639	0.0708 ± 0.0105	HMDB 02329
3	8.244	Butyric acid	0.01684 ± 0.0013	0.01315 ± 0.0019	0.02088 ± 0.025	0.0136 ± 0.0057	0.023 ± 0.0043	HMDB 00357
4	9.466	3-Hydroxybutyric acid	0.0261 ± 0.0112	0.02191 ± 0.003	0.01906 ± 0.0122	0.01847 ± 0.0133	0.0135 ± 0.027	HMDB 00883
5	10.165	Urea	0.74953 ± 0.3128	0.69655 ± 0.1089	1.19208 ± 0.1639	0.84024 ± 0.2563	0.6210 ± 0.0228	HMDB 00294
6	10.756	Phosphoric acid	0.2025 ± 0.0374	0.25623 ± 0.0383	0.30031 ± 0.0406	0.23334 ± 0.0377	0.2436 ± 0.096	HMDB 02142
7	11.283	L-Proline	0.01115 ± 0.0125	0.00575 ± 0.0092	0.01983 ± 0.0202	0.0093 ± 0.0004	0.0083 ± 0.0024	HMDB 00162
8	11.469	Glycine	0.02126 ± 0.0054	0.01324 ± 0.0074	0.01126 ± 0.0134	0.00862 ± 0.0075	0.0724 ± 0.0012	HMDB 00123
9	10.397	Serine	0.00258 ± 0.0023	0.00646 ± 0.0069	0.00137 ± 0.0017	0.00148 ± 0.0019	0.00132 ± 0.0009	HMDB 00187
10	11.185	L-Threonine	0.02325 ± 0.0226	0.02313 ± 0.0046	0.03143 ± 0.0357	0.02225 ± 0.0106	0.0235 ± 0.0109	HMDB 00167
11	14.985	L-Aspartic acid	0.00942 ± 0.002	0.0082 ± 0.0056	0.00485 ± 0.0042	0.00481 ± 0.0068	0.00422 ± 0.0058	HMDB 00191
12	16.033	Erythronic acid	0.1006 ± 0.0172	0.06606 ± 0.0155	0.10925 ± 0.0136	0.05299 ± 0.001	0.06129 ± 0.0034	HMDB 00182
13	18.024	L-Glutamine	0.00405 ± 0.0036	0.0082 ± 0.0078	0.00763 ± 0.0062	0.0088 ± 0.012	0.0062 ± 0.0013	HMDB 00641
14	19.076	Citric acid	0.05004 ± 0.0105	0.02826 ± 0.004	0.02627 ± 0.0077	0.02318 ± 0.0053	0.0136 ± 0.0015	HMDB 00094
15	19.556	N-Acetyl-D-glucosamine	0.01069 ± 0.0094	0.00412 ± 0.0038	0.00769 ± 0.0025	0.00142 ± 0.002	0.0011 ± 0.0001	HMDB 00660
16	20.427	D-Galactose	0.0155 ± 0.0027	0.01657 ± 0.0017	0.01977 ± 0.0087	0.02058 ± 0.0041	0.00188 ± 0.0041	HMDB 00143
17	22.175	D-Mannose	0.01267 ± 0.012	0.01474 ± 0.0128	0.02510 ± 0.0041	0.01819 ± 0.0013	0.0186 ± 0.017	HMDB 00169
18	22.667	Mannitol	0.44126 ± 0.0853	0.43875 ± 0.0532	0.60073 ± 0.1016	0.46482 ± 0.0544	0.5924 ± 0.0454	HMDB 00143
19	22.386	D-Glucose	1.58832 ± 0.1782	1.67471 ± 0.0979	1.27004 ± 0.1011	1.39533 ± 0.208	1.3509 ± 0.1477	HMDB 00122
20	22.803	L-Lysine	0.01849 ± 0.018	0.00986 ± 0.0104	0.0481 ± 0.018	0.01357 ± 0.0078	0.0126 ± 0.0010	HMDB 00182
21	23.076	L-Tyrosine	0.00374 ± 0.0065	0.00357 ± 0.0037	0.02989 ± 0.0305	0.00216 ± 0.0024	0.0219 ± 0.0032	HMDB 00158
22	22.982	D-Turanose	0.00102 ± 0.0018	0.0136 ± 0.0062	0.01904 ± 0.0079	0.0268 ± 0.0038	0.0211 ± 0.0016	HMDB 11740
23	23.734	D-Arabinose	0.02608 ± 0.0115	0.1043 ± 0.0943	0.05253 ± 0.0453	0.09482 ± 0.0859	0.0743 ± 0.0161	HMDB 29942
24	24.877	Hexadecanoic acid	0.3109 ± 0.0146	0.27448 ± 0.0968	0.22018 ± 0.0698	0.2133 ± 0.0317	0.15475 ± 0.0359	HMDB 00220
25	25.411	Myo-inositol	0.02709 ± 0.0011	0.04054 ± 0.0043	0.05513 ± 0.0209	0.03449 ± 0.0027	0.00611 ± 0.0018	HMDB 00211
26	27.025	Linoleic acid	0.09659 ± 0.0158	0.14966 ± 0.0097	0.13681 ± 0.0508	0.10969 ± 0.035	0.1248 ± 0.0049	HMDB 00673
27	27.1	Elaidic acid	0.07306 ± 0.0179	0.10672 ± 0.0386	0.1192 ± 0.0312	0.10621 ± 0.0527	0.1804 ± 0.0036	HMDB 00573
28	27.404	Octadecanoic acid	0.1304 ± 0.0040	0.1216 ± 0.0178	0.10271 ± 0.034	0.10193 ± 0.0115	0.09816 ± 0.018	HMDB 00827
29	28.714	Arachidonic acid	0.0223 ± 0.0016	0.0252 ± 0.0025	0.02653 ± 0.0102	0.0192 ± 0.009	0.0141 ± 0.0018	HMDB 01043
30	32.967	Cholesterol	0.1060 ± 0.0898	0.07909 ± 0.0757	0.0413 ± 0.0837	0.0323 ± 0.0075	0.0451 ± 0.0209	HMDB 00067

## Data Availability

The quality control and metabolomics data used to support the findings of this study are available from the corresponding author upon request.

## Additional Points

*Ethnopharmacological relevance*. Liu-Wei-Di-Huang-Wan (LWDHW) is a classical traditional Chinese formulation, composed of six herbs including *Rehmannia* glutinosa Libosch, *Dioscorea opposita* Thunb, *Cornus officinalis* Sieb. et Zucc, *Poria cocos* (Schw.) Wolf, *Alisma orientalis* (Sam.) Juzep., and *Paeonia suffruticosa* Andr. It has been used widely in China for the treatment and prevention of diabetes and related complications.

## Supplementary Materials

Supplementary Materials Table S1: the linear range for these five compounds. Table S2: identification information of the selected peaks by using HPLC-Q-TOF. Table S3: metabolite information of QC groups. Table S4: result from key metabolic pathway analysis with MetaboAnalyst 3.0. Figure S1: the HPLC chromatography of LWDHW samples and five standards: (1) loganic acid, (2) loganin, (3) 6′-O-galloylpaeoniflorin, (4) benzoylpaeoniflorin, and (5) paeonol. Figure S2: the TIC plots of different groups: blue line: normal control group, red line: MKR model, green line: LWDHW group, and purple line: western medicine group. Figure S3: alanine, aspartate, and glutamate metabolism (impact: 0.7056). Figure S4: linoleic acid metabolism (impact: 0.6562).
