# Supplementary material for: A GC-MS-Based Metabolomics Investigation of the Protective Effect of Liu-Wei-Di-Huang-Wan in Type 2 Diabetes Mellitus Mice
Source: Int J Anal Chem. 2020 Aug 13;2020:1306439. doi: 10.1155/2020/1306439 (PMC7443003; doi:10.1155/2020/1306439)
Supplement: Supplementary Materials — Table S1: the linear range for these five compounds. Table S2: identification information of the selected peaks by using HPLC-Q-TOF. Table S3: metabolite information of QC groups. Table S4: result from key metabolic pathway analysis with MetaboAnalyst 3.0. Figure S1: the HPLC chromatography of LWDHW samples and five standards: (1) loganic acid, (2) loganin, (3) 6′-O-galloylpaeoniflorin, (4) benzoylpaeoniflorin, and (5) paeonol. Figure S2: the TIC plots of different groups: blue line: normal control group, red line: MKR model, green line: LWDHW group, and purple line: western medicine group. Figure S3: alanine, aspartate, and glutamate metabolism (impact: 0.7056). Figure S4: linoleic acid metabolism (impact: 0.6562). [file 1306439.f1.docx]

A *GC-MS-based* metabolomics investigation of the protective effect of Liu-Wei-Di-Huang-Wan in type 2 diabetes mellitus mice

Jian-hua Huang ^1ξ,3^, Dan He^ξ1,2^, Lin Chen^1^,Qing Du^,2^, Ping Cai^1^, Rong Yu^1,2*^, Shui-han Zhang ^1,2*^

^[[1]](#footnote-1)^1. Hunan Key Laboratory of TCM Prescription and Syndromes Translational Medicine; Hunan University of Chinese Medicine, Changsha, Hunan, 410208, P. R. China;

2. Hunan academy of Chinese Medicine, Changsha, Hunan, 410013, P. R. China;

3. Hunan University of Chinese Medicine, Changsha, Hunan, 410208, P. R. China;

Table S1 The linear range for these five compounds.

| **Compounds** | **Linear** | **Relationship** | **Ranges（μg)** |
| --- | --- | --- | --- |
| Paeonol | y = 8223x + 51.626 | R² = 0.9998 | 0.1096~1.096 |
| 6'-O-Galloylpaeoniflorin | y = 3011.9x + 2.9423 | R² = 0.9991 | 0.01196~0.1196 |
| Benzoylpaeoniflorin | y = 6388.2x + 8.1588 | R² = 0.9999 | 0.025~0.375 |
| Loganic acid | y = 4316x + 2.7007 | R² = 1 | 0.0213~0.213 |
| Loganin | y = 4936.9x + 28.778 | R² = 0.9998 | 0.0788~1.182 |

Table S2: Identification information of the selected peaks by using HPLC-Q-TOF

| No. | t_R_（min) | ion | Experimental mass(m/z) | Theoretical mass(m/z) | Mass error(ppm) | Formula | Structural identification | Original |
| --- | --- | --- | --- | --- | --- | --- | --- | --- |
| 1 | 15.060 | [M-H]^-^ | 375.1276 | 375.1297 | 5.60 | C_16_H_24_O_10_ | Loganic acid | *Fructus Corni* |
| 2 | 19.693 | [M+COOH]^-^ | 435.1521 | 435.1508 | 2.98 | C_17_H_26_O_10_ | Loganin | *Fructus Corni* |
| 3 | 26.933 | [M-H]^-^ | 631.1675 | 631.1668 | 1.19 | C_30_H_32_O_15_ | 6'-O-Galloylpaeoniflorin | *Cortex Moutan* |
| 4 | 35.200 | [M+COOH]^-^ | 629.1898 | 629.1876 | 3.49 | C_30_H_32_O_12_ | Benzoylpaeoniflorin | *Cortex Moutan* |
| 5 | 38.387 | [M-H]^-^ | 165.0548 | 165.0557 | 5.45 | C_9_H_10_O_3_ | Paeonol | *Fructus Corni, Cortex Moutan* |

Table S3: Metabolite information of QC groups.

| id | RT (min) | Endogenous  metabolites | QC samples | |
| --- | --- | --- | --- | --- |
|  |  |  | Concentrations | RSD (%) |
| 1 | 6.563 | Oxalic acid | 1.0566±0.03925 | 3.71 |
| 2 | 7.329 | L-Lactic acid | 0.09105±0.0030 | 3.29 |
| 3 | 8.244 | Butyric acid | 0.0150±0.00061 | 4.06 |
| 4 | 9.466 | 3-Hydroxybutyric acid | 0.01877±0.0163 | 5.13 |
| 5 | 10.165 | Urea | 0.7230±0.2108 | 8.68 |
| 6 | 10.756 | Phosphoric acid | 0.2294±0.0178 | 7.75 |
| 7 | 11.283 | L-Proline | 0.01045±0.0011 | 10.02 |
| 8 | 11.469 | Glycine | 0.01725±0.0024 | 12.30 |
| 9 | 10.397 | Serine | 0.00452±0.006 | 1.32 |
| 10 | 11.185 | L-Threonine | 0.02125±0.0121 | 5.73 |
| 11 | 14.985 | L-Aspartic acid | 0.0071±0.0006 | 8.68 |
| 12 | 16.033 | Erythronic acid | 0.0833±0.00778 | 9.35 |
| 13 | 18.024 | L-Glutamine | 0.0161±0.0011 | 6.96 |
| 14 | 19.076 | Citric acid | 0.02473±0.0032 | 1.31 |
| 15 | 19.556 | N-acetyl-D-glucosamine | 0.0074±0.0026 | 3.52 |
| 16 | 20.427 | D-Galactose | 0.00120±0.0005 | 4.11 |
| 17 | 22.175 | D-mannose | 0.0137±0.0055 | 4.03 |
| 18 | 22.667 | Mannitol | 0.4505±0.0166 | 3.69 |
| 19 | 22.386 | D-Glucose | 1.5315±0.1380 | 5.88 |
| 20 | 22.803 | L-Lysine | 0.01417±0.0107 | 7.62 |
| 21 | 23.076 | L-Tyrosine | 0.00365±0.0043 | 11.32 |
| 22 | 22.982 | D-Turanose | 0.00231±0.0019 | 8.42 |
| 23 | 23.734 | D-Arabinose | 0.06519±0.037 | 5.65 |
| 24 | 24.877 | Hexadecanoic acid | 0.1840±0.0094 | 5.13 |
| 25 | 25.411 | Myo-Inositol | 0.0338±0.0030 | 8.7 |
| 26 | 27.025 | Linoleic acid | 0.1243±0.060 | 4.90 |
| 27 | 27.1 | Elaidic acid | 0.0898±0.0058 | 6.40 |
| 28 | 27.404 | Octadecanoic acid | 0.1098±0.0065 | 5.92 |
| 29 | 28.714 | Arachidonic acid | 0.02375±0.0089 | 3.7 |
| 30 | 32.967 | Cholesterol | 0.1198±0.0062 | 5.19 |

**Table S4.** Result from key Metabolic Pathway Analysis with MetaboAnalyst 3.0

| **No.** | **Pathway name** | **total cmpd** | **hits** | **raw p** | **-log (p)** | **impact** |
| --- | --- | --- | --- | --- | --- | --- |
| 1 | Alanine, aspartate and glutamate metabolism | 24 | 4 | 0.00040342 | 7.8155 | 0.7056 |
| 2 | Linoleic acid metabolism | 15 | 1 | 0.20789 | 1.5708 | 0.65625 |
| 3 | Arginine and proline metabolism | 77 | 6 | 0.0009288 | 6.9815 | 0.1749 |
| 4 | Aminoacyl-tRNA biosynthesis | 75 | 13 | 1.77E-11 | 24.755 | 0.2253 |
| 5 | Glycine, serine and threonine metabolism | 48 | 4 | 0.005674 | 5.1719 | 0.4203 |
| 6 | Phenylalanine metabolism | 45 | 2 | 0.15102 | 1.8904 | 0.1192 |
| 7 | Arachidonic acid metabolism | 62 | 1 | 0.62203 | 0.47477 | 0.2166 |


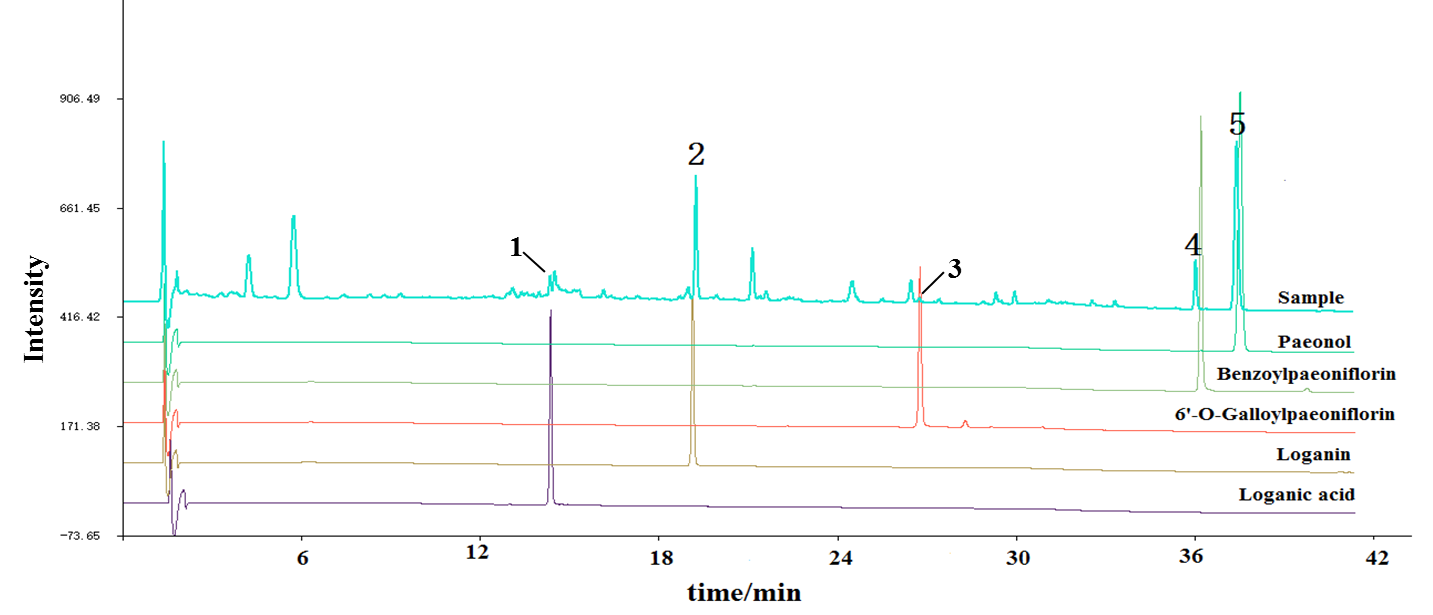
Figure S1:The HPLC chromatraghy of LWDHW samples and five standards. 1: Loganic acid, 2: Loganin, 3: 6'-O-Galloylpaeoniflorin,4: Benzoylpaeoniflorin, 5: Paeonol.


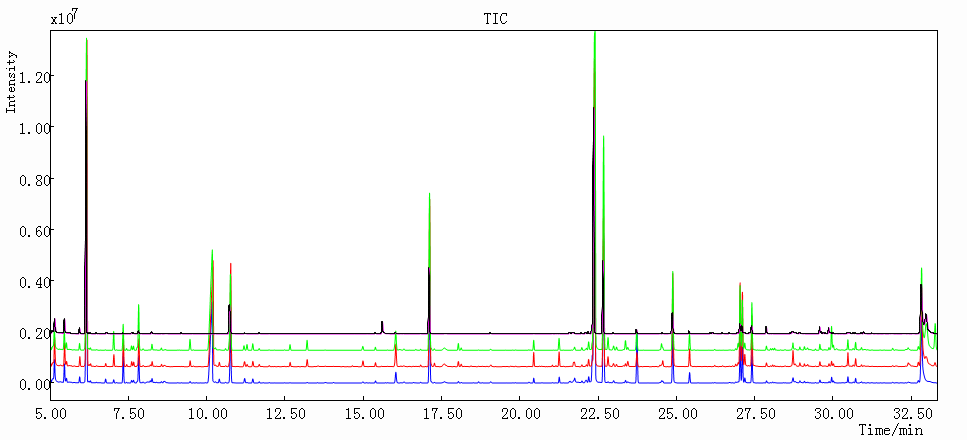


Figure S2：The TIC plots of different groups Blue line: Normal control grouop, Red line: MKR model, Green line: LWDHW group, Purple line: West medicine group.


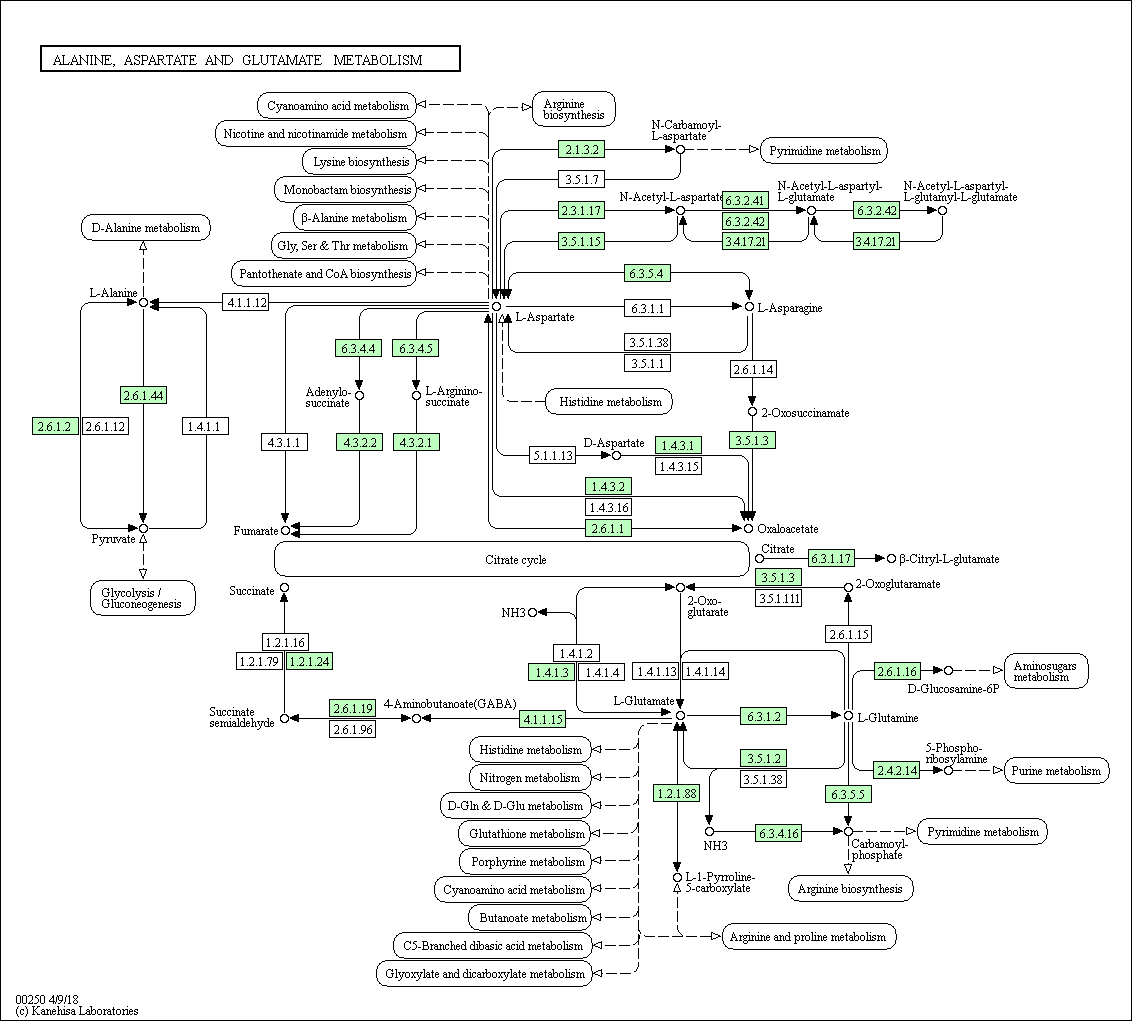


**Figure S3.** Alanine, aspartate and glutamate metabolism (impact: 0.7056)


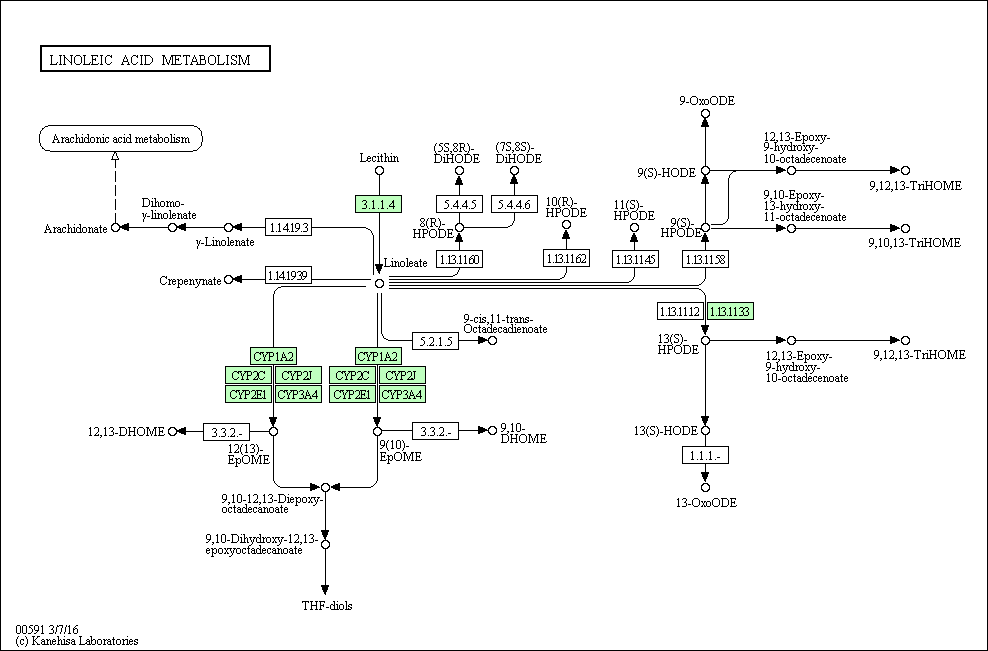


**Figure S4.** Linoleic acid metabolism (impact: 0.6562)

1. * Corresponding author at: Hunan Key Laboratory of TCM Prescription and Syndromes Translational Medicine; Hunan University of Chinese Medicine, Changsha, Hunan, 410208, P. R. China;

   Correspondence to: [1208466238@qq.com](mailto:1208466238@qq.com) (Rong Yu); [zhangshuihan0220@126.com](mailto:zhangshuihan0220@126.com) (shui-han Zhang)

   ξ The first two authors have equal contribution to this article. [↑](#footnote-ref-1)
